# 708. Three Stages of Laboratory Stewardship in Improving Appropriate *Clostridiodes Difficile* Testing

**DOI:** 10.1093/ofid/ofad500.770

**Published:** 2023-11-27

**Authors:** Shannon Beckman, Gretchen Zimmerman, Khateeb Raza, Samuel Ballard, Naveen Vijayam, Bethany Stibbe, Monica Rykse, Joe Brown, Theresa Klein, Andrew M Skinner, Michael S Wang

**Affiliations:** Corewell Health Lakeland, Saint Joseph, Michigan; Corewell Health Lakeland, Saint Joseph, Michigan; Corewell Health Lakeland, Saint Joseph, Michigan; Corewell Health Lakeland, Saint Joseph, Michigan; Corewell Health Lakeland, Saint Joseph, Michigan; Corewell Health Lakeland, Saint Joseph, Michigan; Corewell Health Lakeland, Saint Joseph, Michigan; Corewell Health Lakeland, Saint Joseph, Michigan; Corewell Health Lakeland, Saint Joseph, Michigan; University of Utah, Holladay, Utah; Spectrum Health Lakeland, St Joseph, Michigan

## Abstract

**Background:**

Clostridioides difficile infections are a frequent cause of nosocomial infections and a large financial burden to the health care system. Molecular diagnostics such as PCR may frequently detect colonized patients and lead to overdiagnosis. Using the electronic medical record (EMR) system, we implemented multiple interventions to improve testing stewardship.

**Methods:**

Over the span of 3 years, our Infection Control (IC) team implemented 3 separate testing interventions: in July 2019, a hard stop was implemented, requiring providers to document >3 loose or watery stools and no laxative use within 48 hours. In July 2020, Stage 2 was implemented, requiring an infectious diseases (ID) physician to electronically approve a CDI test for all tests after 3 midnights. In April 2022, Stage 3 was implemented, changing the testing protocol to an algorithmic approach, with positive C. difficile PCR reflexing to a toxin immunoassay with regular surveillance by IC practitioners to ensure compliance.

**Results:**

From 2019 to 2022, our CDI testing interventions resulted in a reduction of C. difficile tests completed from 21.22 tests/1000 patient days (PD) to 6.95 tests/1000 PD (p< 0.01). The median number of tests completed in persons admitted for ≤ 3 days decreased from 63 (Interquartile Range (IQR):61 – 67) to 28 (IQR:26-35), p< 0.01. The median number of tests completed in persons admitted for > 3 days decreased from 34 (IQR:31 – 35) to 9 (IQR:7-10), p< 0.01. and the median, decrease of 27.8% (36 to 26), and increase of 7.69% (26 to 28), p < 0.01, respectively. The PCR percent positivity remained constant after the 1st intervention (15.3% vs. 14.4%, p=NS) but there was a slight increase in the test percent positivity between intervention 1 and 2 (19.2%, p=.08). After the 3rd intervention, the PCR percent positivity was 20.2% (80/316). However, the majority of these were PCR positive/toxin negative (74% vs 26%, p< 0.01).

**Conclusion:**

3 stages of EMR-based interventions, including testing indications, ID physician review, and 2-tiered testing plus IC review, were effective in reducing CDI tests. These interventions led to an upward trend in percent positivity indicating that persons appropriate for testing were not adversely impacted, but further study is necessary to determine the long-term impacts on patient outcomes.

**Disclosures:**

**Andrew M. Skinner, MD**, Academy for Continued Healthcare Learning: Honoraria|American Society of Healthcare Pharmacists: Honoraria|Ferring Pharmaceuticals: Honoraria|MJH Life Sciences: Honoraria

